# Light-driven bacterial inactivation using *Plumeria rubra* extract: a promising natural photosensitizer

**DOI:** 10.1007/s10123-026-00818-z

**Published:** 2026-04-10

**Authors:** Caroline T. Marcon, Leandro S. Herculano, Tânia C. S. P. Pires, Cristiane Canan, Mônica L. Fiorese, Luis C. Malacarne, Tayse F. F. Silveira, Joana A. S. Amaral, Lillian B. Barros

**Affiliations:** 1 Programa de Pós-graduação em Engenharia Química, Universidade Estadual do Oeste do Paraná, R. Guaíra 3141, Toledo, 85903-000 Paraná Brasil; 2 Departamento de Física, Universidade Tecnológica Federal do Paraná, Av. Brasil 4232, Medianeira, 85722-332 Paraná Brasil; 3 Research Centre for Active Living and Wellbeing (LiveWell), Instituto Politécnico de Bragança, Campus de Santa Apolónia, Bragança, 5300-253 Portugal; 4 Departamento de Alimentos, Universidade Tecnológica Federal do Paraná, Av. Brasil 4232, Medianeira, 85722-332 Paraná Brasil; 5 Departamento de Física, Universidade Estadual de Maringá, Av. Colombo 5790, Maringá, 87020-900 Paraná Brasil; 6 Centro de Investigação de Montanha, SusTEC, Instituto Politécnico de Bragança, Campus de Santa Apolónia, Bragança, 5300-253 Portugal

**Keywords:** Non-conventional edible plants, Antimicrobial photodynamic therapy, Plant-derived compounds, Bioactive extracts

## Abstract

Antimicrobial Photodynamic Inactivation (aPDI) is a non-thermal, minimally invasive technique that uses light and photosensitizing compounds (PS) to generate reactive oxygen species, promoting the destruction of microorganisms without inducing microbial resistance. This study investigated the photosensitizing potential of the flower extract of *Plumeria rubra* L., a unconventional edible plant, in aPDI. The extract showed a high content of rutin, a flavonoid known to confer chemical stability to PS and promote its interaction with bacterial cell structures, increasing photodynamic efficiency. Tests conducted in the dark revealed a lack of antimicrobial activity, a desirable characteristic for photosensitizing compounds. Under white light irradiation and an optical fluence of 18 ${\textbf {J}}/{\textbf {cm}}^{\boldsymbol{2}}$, the extract inactivated the methicillin-resistant *Staphylococcus aureus* strain at a concentration of 0.78 mg/mL, in addition to demonstrating action against *Listeria monocytogenes* and *Staphylococcus epidermidis*. At an optical fluence of 27 ${\textbf {J}}/{\textbf {cm}}^{\boldsymbol{2}}$, inactivation of most of the strains evaluated was observed. These results indicate that *P. rubra* extract it is a promising natural photosensitizer, that combines a favorable phytochemical composition, absence of inactivation in the dark, and high photodynamic efficiency under white light, demonstrating potential for therapeutic and industrial applications.

## Introduction

Photodynamic inactivation (PDI) can be understood as a non-thermal and minimally invasive technique that uses light and photosensitizing compounds (PS) to generate reactive oxygen species (ROS) that interact with cells and microorganisms, leading to their death. It emerges as an auxiliary or alternative technique to traditional preservation or treatment methods, offering broad-spectrum antimicrobial activity that does not generate microbial resistance (Henderson and Dougherty 1992; Dougherty et al. 1998; Castano et al. 2004; Hamblin 2016; Pang et al. 2025).

PDI is based on three main pillars: a non-toxic compound that is activated by light (PS); a light source with an emission wavelength that matches the optical absorption band of PS; and the presence of oxygen in the target tissue or cellular environment (Dougherty et al. 1998; Castano et al. 2004).

**Fig. 1:**
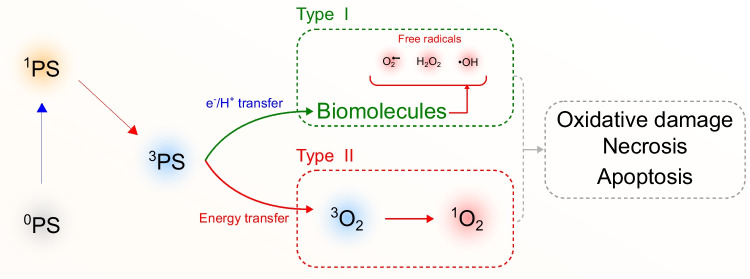
Simplified representation of the mechanism of action of PDI: Light absorption by $^0\textrm{PS}$ and transition from the $^1\textrm{PS}$ state, decay by intersystem crossing to the $^3\textrm{PS}$ state, followed by two paths: transfer of electrons or hydrogen to biomolecules generating free radicals (Type I reaction) or energy transfer from $^3\textrm{PS}$ to $^3\textrm{O}_{2}$ generating $^1\textrm{O}_{2}$ (Type II reaction), in both the ROS generated causes cell death

The PDI mechanism of action begins with the absorption of light by PS in the ground singlet state ($^{\textrm{0PS}}$), which transitions to the excited and unstable singlet state ($^1$PS). Through intersystem crossing, $^1$PS converts to the triplet state ($^3$PS), which has a longer lifetime. From this point on, the PDI mechanism can follow two main reactions that generate ROS. In the Type I reaction, $^3$PS reacts with substances present in the surrounding medium via electron or hydrogen transfer, resulting in the formation of free radicals, including superoxide anion, hydrogen peroxide, and hydroxyl radicals, which cause oxidative damage to cells. In the Type II reaction, $^3$PS transfers energy directly to oxygen in the triplet state ($^3\textrm{O}_{2}$), converting it to the highly reactive singlet state ($^1\textrm{O}_{2}$), which, like other ROS, generates cellular damage and induces cell death. Figure 1 presents the aPDI mechanism through a simplified diagram (Henderson and Dougherty 1992; Dougherty et al. 1998; Castano et al. 2004; Hamblin 2016).

PDI, including the antimicrobial modality (aPDI), is a minimally invasive approach with broad and expanding applications. They are considered a relevant strategy against bacterial resistance, also demonstrating efficacy in inactivating some fungi, viruses, parasites, and microbial biofilms. When used to combat disease, it is more commonly referred to as Photodynamic Therapy (PDT). It has been used in oncology for the treatment of solid tumors and precancerous lesions, in ophthalmology for the treatment of retinal macular degeneration, in dermatology for reducing acne and fungal infections, and in dentistry for oral disinfection and the treatment of periodontitis (Hamblin 2016; Nayak et al. 2025; Fiegler-Rudol et al. 2025; Pang et al. 2025; Qureshi et al. 2024).

PDI/aPDI/PDT is a promising therapeutic approach, in which the efficacy depends heavily on the appropriate selection of PS. Conventional synthetic PS, such as first- and second-generation porphyrins, have clinical limitations due to toxicity, limited availability, and difficulty in controlling cellular damage. In this context, it is essential to explore PS of natural origin, recognized for their safety, low toxicity, abundance, and high biocompatibility (Aziz et al. 2023; Wang et al. 2023).

The structural diversity of natural PS confers selectivity and specific biological activities, making them promising alternatives that may be more effective than synthetic analogues. Therefore, the investigation of natural PS constitutes a strategic avenue for improving aPDI/PDT, offering safer and more sustainable therapies that can address clinical challenges such as antimicrobial resistance (Aziz et al. 2023; Wang et al. 2023; Aebisher et al. 2024; Zhou et al. 2024).

Unconventional Edible Plants (UEP) are a viable alternative source to contribute to the search for new natural PS. UEP are commercially underexploited edible plant species that are not part of the usual diet but possess nutritional, functional, or bioactive value. The exploitation of PS in UEP offers several advantages: they can provide natural pigments such as anthocyanins, carotenoids, chlorophylls, and betalains, which, in addition to imparting color to foods, cosmetics, or therapeutic products, often exhibit biological activities such as antioxidant, antimicrobial, or photosensitizing action. Bioprospecting these plants also adds economic value to underutilized species, encourages the cultivation and preservation of local biodiversity, and enables the development of innovative functional products that combine nutritional, therapeutic, and environmental benefits in a single natural resource (Leal et al. 2018; Milião et al. 2022; Ferreira et al. 2024).

In this work, we report the methodology and results of evaluating the extract from the flowers of UEP *Plumeria rubra* L. (*P. rubra*) as a candidate for PS in aPDI for the elimination of bacteria causing foodborne diseases and of clinical interest.

*P. rubra* (Family: Apocynaceae) is native to Central America, northern South America, and Mexico, and is now widely distributed in regions such as the Caribbean, South America, Africa, China, and the Pacific. It is an easy-to-grow plant that can thrive in a variety of soil types, including clay and sandy soils, as well as acidic and alkaline conditions. However, it performs best in well-drained soils. Gilman and Watson (1994); Vahl (2012); Acevedo-Rodríguez and Strong (2012).

**Fig. 2:**
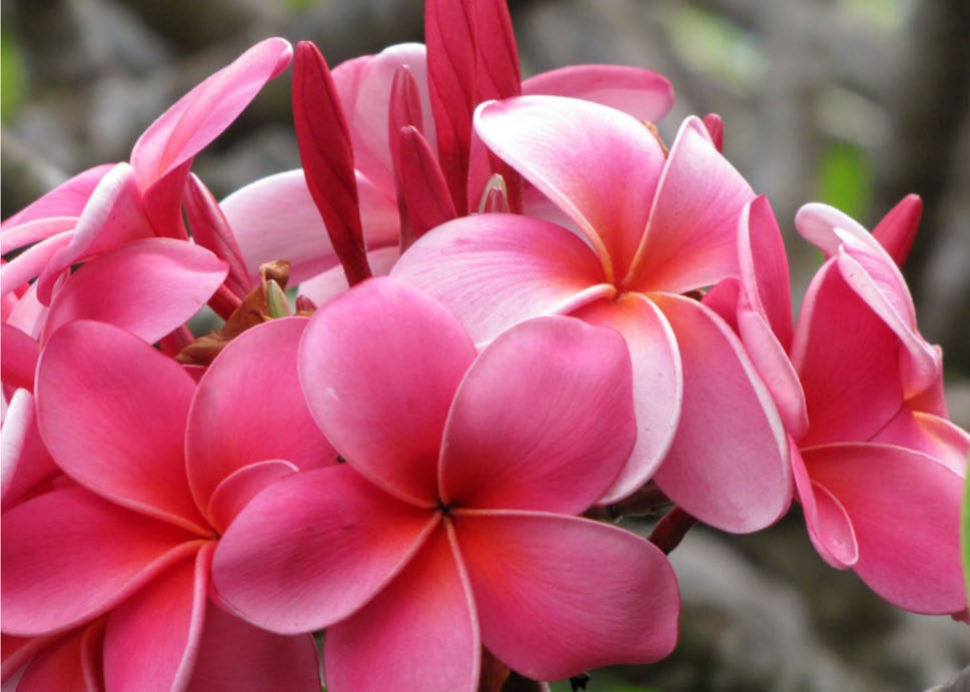
Photograph of the flowers of the *P. rubra*. Image reproduced from Rojas-Sandoval, CC BY-SA 3.0 license (Rojas-Sandoval 2024)

*P. rubra* is widely cultivated as an ornamental plant in gardens and public spaces, characterized by salpiform flowers with variable colors, including white, yellow, pink, red, or spots with shades Fig. 2 shows a photograph of *P. rubra* flowers. Gilman and Watson (1994); Rojas-Sandoval (2024)

*P. rubra *exhibits phytochemical diversity marked by anthocyanins, iridoids, triterpenes, flavonoids, as well as phenolic acids, coumarins, aromatic esters, and phytosterols. This composition reflects the bioactive potential of this specie, associated with specific anti-inflammatory, antioxidant, and pharmacological properties. Byamukama et al. (n.d.); Sharma et al. (2011); Isa et al. (2018); Bihani (2021); Malaspina et al. (2024); Calva and Celi (2024)

Widely used in traditional medicine in pantropical regions, *P. rubra* has diverse therapeutic applications. In South America, decoctions of leaves, bark, and heartwood are used as anthelmintics, purgatives, and emmenagogues, as well as in topical preparations for skin diseases. In East Asia, the fruit pulp, latex, and stem bark are traditionally used as abortifacients and purgatives. In addition to its medicinal value, the flowers of this species are used in the preparation of sweets, jellies, and salads (Hamburger et al. 1991; Sharma et al. 2011; Kalantri and Aher 2016).

To evaluate the potential of *P. rubra* flowers to provide a natural PS, the flower extract was obtained via ultrasound-assisted alcoholic extraction, followed by the characterization of the phenolic compound profile, antioxidant activity, antibacterial activity, light absorption in the visible region of the electromagnetic spectrum and capacity for photodynamic bacterial inactivation in vitro, determining the light dosimetry and concentration required for this purpose.

## Materials and methods

### Obtaining the extract

Fresh flowers of *P. rubra* were collected by hand, cleaned with distilled water to remove impurities, and dried in an oven with air circulation at 40–45 $^\circ$C for approximately 48 hours, until they reached a constant weight. The dried flowers were then ground in a knife mill to a fine powder. The material was stored in vacuum-sealed plastic bags at a temperature of -18 $^\circ$C until the extract was obtained.

The aqueous extract of *P. rubra* flowers was obtained by hydroalcoholic extraction (70% v/v) assisted by ultrasound (Elma, Elmasonic P) at a frequency of 80 kHz and a power of 100 W for 60 minutes. Subsequently, the extracts were concentrated in a rotary evaporator (Fisatom, Model 804) until reaching approximately 20% of the initial volume and then lyophilized (Labconco, Freeze Zone 6) at a temperature of -50 $^\circ$C and an absolute pressure of less than 0.8 mBar, undergoing a heat exchange process with a heating source at 40 $^\circ$C for 40 h or until completely dry. The dyes were packaged in hermetically sealed jars and stored at 4 $^\circ$C for future analysis.

The choice of the ultrasound-assisted hydroalcoholic solution extraction method was based on the advantages associated with this technique. Ethanol, used as a solvent, is considered safe, non-toxic, easily obtained, comes from a renewable source, and has a high affinity for phenolic compounds. Furthermore, ultrasound-assisted extraction, due to the phenomenon of acoustic cavitation, generates shock waves capable of rupturing the cell walls of the plant material. This process increases the contact area between the matrix and the solvent, thereby favoring the diffusion of the target compounds and enabling extraction in less time, at moderate temperatures, with reduced energy and solvent consumption (Kumar et al. 2021; Cui et al. 2022; Silva et al. 2023; Athirojthanakij and Rashidinejad 2024; Sethi and Rathod 2025).

### Phenolic profile

Phenolic compounds were analyzed using a UHPLC system (Thermo Scientific, Dionex Ultimate 3000) equipped with a diode array detector coupled in series to a high-resolution mass spectrometer (Orbitrap Exploris 120, Thermo Finnigan). Separation was performed on a Luna C18 column (3 $\mathrm {\mu }$m *×* 4.6 *×* 150 mm) maintained at 35 $^\circ$C (Phenomenex, Torrance). Chromatographic conditions used for compound separation were established as described in detail by Gonçalves et al. (2024).

For the mass spectrometer, heated electrospray ionization (H-ESI) in negative mode (2500 V) was employed. Nebulizing gas ($\textrm{N}_{2}$) was supplied at a flow rate of 50 a.u., with auxiliary gas at 10 a.u. and sweep gas at one a.u. The ion transfer tube and vaporizer temperatures were set at 325 $^\circ$C and 350 $^\circ$C, respectively. Data-dependent acquisition mode was used with a normalized collision energy of 30%. Mass spectra were recorded in the m/z range of 110–1100, with a resolution of 60,000 for MS and 17,000 for MS/MS.

Compound identification was based on UV–Vis spectra, accurate mass, molecular formula, and fragmentation pattern, compared with authentic standards or literature data. Quantification of phenolic compounds was performed using external calibration curves of the respective standards or, when unavailable, of the most structurally similar compound.

### Antioxidant activity

A PS with high antioxidant activity in the absence of light can exhibit greater chemical stability and also contribute to reducing its basal toxicity (in the dark), thereby minimizing potential side effects on tissues before light application. Based on this, the extract from *P. rubra* flowers was subjected to the Thiobarbituric Acid Reactive Substances (TBARS) assay to evaluate its antioxidant activity.

The TBARS assay was adapted to evaluate the antioxidant potential of *P. rubra* flower extract. The method is based on the reaction between lipid peroxidation products, such as malondialdehyde (MDA), and thiobarbituric acid (TBA), forming a complex that can be quantified spectrophotometrically (Ghani et al. 2017; Christodoulou et al. 2022).

The experimental procedure used porcine brain homogenate in Tris-HCl buffer, to which ascorbic acid (0.1 mM, 0.1 mL), ferrous sulfate (10 *μ M*, 0.1 mL), and different concentrations of *P. rubra* extract were added. After incubation at 37 $^\circ$C to induce peroxidation, trichloroacetic (28 % w/v, 0.5 mL) acid and TBA (2% w/v, 0.38 mL) were added, followed by further incubation at 80 $^\circ$C. The samples were then centrifuged, and the supernatant was analyzed for absorbance at 532 nm.

The analysis of antioxidant activity, as measured by the TBARS assay, was performed by relating the absorbance at 532 nm to the extract concentration, using a logistic model to adjust the data, as described in Eq. 1.

1 $$\begin{aligned} A_{532}(C) = A_2+\dfrac{\left( A_1 - A_2\right) }{\left[ 1+\left( \dfrac{C}{EC_{50}}\right) ^p\right] }. \end{aligned}$$

Applied to the TBARS technique, the parameters of the logistic model, given by Eq. 1, can be described as: $\textrm{A}_{1}$ being the absorbance value without extract, such that oxidation is maximum, that is, without antioxidant action; $\textrm{A}_{2}$ being the absorbance at a high concentration of the extract, corresponding to minimum oxidation; $\textrm{EC}_{50}$ being the concentration that reduces oxidation by 50%, that is, the lower the $\textrm{EC}_{50}$, the greater the antioxidant action of the extract; and the parameter $\textrm{p}$, known as the Hill coefficient, here denoted as the slope parameter and which indicates how oxidation inhibition evolves with extract concentration.

### Antibacterial activity

It is desirable that the PS does not exhibit antimicrobial activity in the absence of light, or that the minimum inhibitory concentration (MIC) and the minimum bactericidal concentration (MBC) values are high under these conditions. Before performing the aPDI assay, the antimicrobial activity of the candidate PS was evaluated in the absence of light. For this purpose, MIC and MBC were determined and also served as references to establish the concentrations of the *P. rubra* flower extract used in the aPDI assay.

The efficacy of *P. rubra* extract, both in the antimicrobial activity assay and in the aPDI assay, was evaluated against the following bacteria: *Cutibacterium acnes* ATCC 11827 (*C. acnes*), clinically isolated *Enterococcus faecalis* (*E. faecalis* CI), *Listeria monocytogene*s ATCC 19111 (*L. monocytogenes*) and clinically isolated *Listeria monocytogenes* (*L. monocytogenes* CI), clinically isolated *Morganella morganii* (*M. morganii* CI), clinically isolated methicillin-resistant *Staphylococcus aureus* (MRSA CI), *Pseudomonas aeruginosa* ATCC 9027 (*P. aeruginosa*), *Staphylococcus aureus* ATCC 25923 (*S. aureus*) and *Staphylococcus epidermidis* ATCC 12228 (*S. epidermidis*).

The bacterial strains were cultured in Petri dishes and incubated for 24 h at 37 $^\circ$C to obtain viable bacteria at the maximum growth rate (*C. acnes* and *S. epidermidis* were incubated under anaerobic conditions). Then, using a McFarland densitometer, 10 mL of inocula were obtained in Muller-Hinton medium containing 10$^6$ CFU/mL. These inocula were placed in a 96-well plate containing different concentrations (obtained by serial dilution) of *P. rubra* flower extract (from 100 mg/mL to 0.1 mg/mL), totaling 90 $\mu \textrm{L}$ per well. Subsequently, 10 *μ*L of the inoculum was added to each well to obtain a concentration of 10$^5$ CFU/mL. The plates were incubated at 37 $^\circ$C for 24 hours.

After the incubation period, 100 $\mu \textrm{L}$ of an aqueous solution containing 0.5 mg/mL of iodonitrotetrazolium (INT), which acted as a revealing agent for the bacterial metabolic activity, was added to each well. After four hours, it was verified that the wells containing the lowest concentration of the *P. rubra* extract did not show a color change, indicating the MIC. An aliquot of 10 $\mu \textrm{L}$ from the remaining wells, which also showed no change in color, was transferred to Petri dishes containing solid Muller-Hinton medium and incubated at 37 $^\circ$C for 24 h to determine the MBC.

### UV-VIS spectroscopy

To determine the ideal light source for the aPDI assay, the absorbance spectra of the *P. rubra* flower extract diluted in water were determined as a function of concentration. This analysis was performed using a spectrophotometer capable of reading in 96-well plates (SpectroStar Nano, BMG Labtec). Starting from a concentration of 30 mg/mL and using the serial dilution procedure in a 96-well plate, spectra were acquired in the 400-700 nm region, with a 2 nm interval, for the aqueous extract at concentrations ranging from 30.00 mg/mL to 0.23 mg/mL (eight concentrations).

### Light source

To ensure uniform light intensity across the surface of a 96-well plate, an LED-based lighting system was developed. The system consisted of 20 LEDs distributed evenly across an aluminum printed circuit board (PCB), coupled with a forced ventilation system to optimize heat dissipation.

The PCB-LED array was positioned on the underside of a rectangular tube whose inner walls were lined with mirrored acrylic sheets to increase efficiency and direct light emission to the top surface of the plate. A sandblasted acrylic sheet measuring 130 *×* 90 mm was placed on top to promote uniform light distribution. All components were integrated into a black acrylic housing.

The system’s irradiation intensity (W/cm$^2$) was regulated by a microcontroller and calibrated using a spectroradiometer (Gooch & Housego; OL 756) coupled to an optical fiber (Gooch & Housego, OL 730 7q-1.0) and a 50.8 mm diameter integrating sphere (Gooch & Housego, IS-270).

### Antimicrobial photodynamic inactivation assay

The aPDI assay was performed, as previously described (Herculano et al. 2026), to determine the MBC of *P. rubra* flower extract for different bacteria and under different optical fluences. To this end, bacterial inocula were prepared in advance to provide an aqueous solution containing $10^5$ CFU/mL.

Using a 96-well flat-bottom plate, 150 $\mu \textrm{L}$ of sterile water was added to the wells of rows B through H and columns 1 through 3 (in triplicate). Then, 150 $\mu \textrm{L}$ of an aqueous solution containing 50.00 mg/mL of *P. rubra* extract was added to the wells of rows A and B. A serial dilution was then performed by transferring 150 $\mu \textrm{L}$ from the wells of row B to the wells of row C, and this process was repeated successively up to row G. The wells of row H were kept free of extract to verify the effect of light on bacterial multiplication.

Continuing the assay, 15 $\mu \textrm{L}$ of the bacterial inoculum was added, resulting in approximately 10$^5$ CFU/mL in each well. After inoculation, the 96-well plate was kept in the dark for 15 min to allow the extract to interact with the bacteria.

Next, to determine the effect of the extract in the absence of light, 3 $\mu \textrm{L}$ from each well was transferred to a Petri dish containing solid medium (PCA agar) using an eight-channel micropipette to obtain three vertical lines with eight pipetting points. An arrow was drawn on the bottom of the Petri dish to indicate the direction of increasing extract concentration. The plate was kept in a laminar flow hood to dry and then transferred to incubation at 37 $^\circ$C for 24 h.

To evaluate the effect of the extract on bacteria when exposed to light, i.e., photodynamic inactivation, the same 96-well plate was subjected to illumination using the previously described light source for 15 minutes. After this illumination period, 3 $\mu \textrm{L}$ from each well was transferred to a new Petri dish containing solid medium, using an eight-channel micropipette, to obtain three vertical lines with eight pipetting points each. An arrow was drawn at the bottom of the Petri dish to indicate the direction of increasing extract concentration. The Petri dish was kept in a laminar flow chamber for drying and then incubated at 37 $^\circ$C for 24 h.

The same plate was illuminated again for the same time interval. The process of transferring 3 $\mu \textrm{L}$ to a Petri dish, drying, and incubation was repeated, resulting in 4 Petri dishes associated with exposure times of 0, 15, 30, and 45 min, corresponding to optical fluence values of 0, 9, 18, and 27 $\mathrm {J/cm}^{2}$. The entire process described above was repeated for each bacterium separately.

After the Petri dish incubation period, the Petri dishes were photographed, and their images were organized and cropped to show the area of interest (inside the dish) using the vector drawing software Inkscape.

**Fig. 3:**
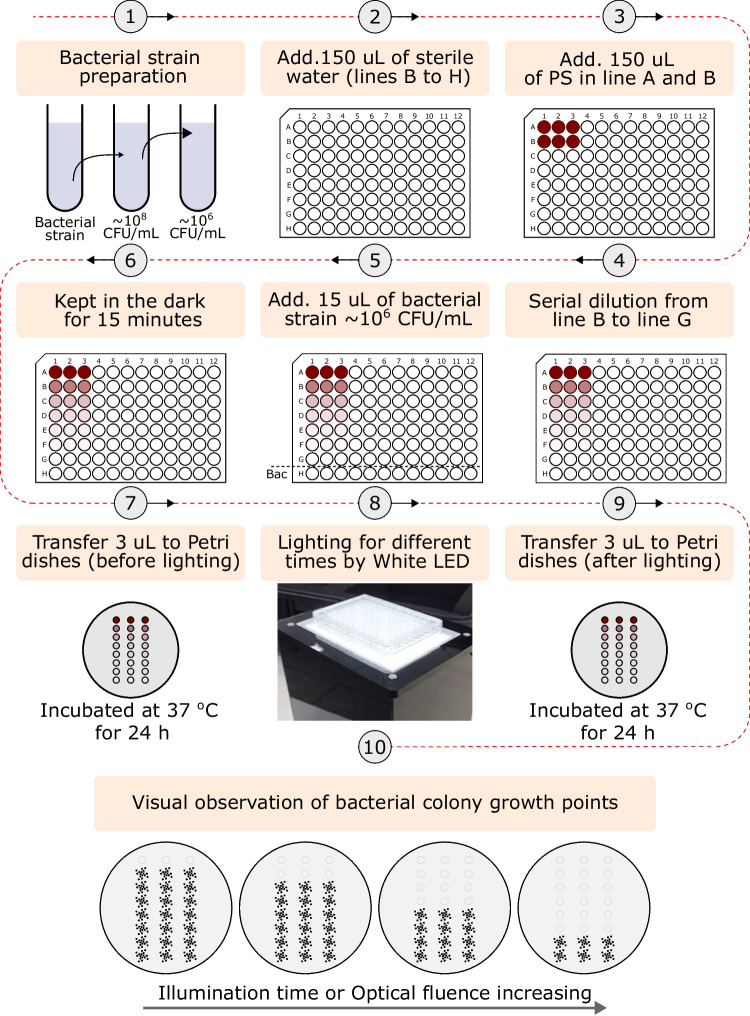
Schematic of the aPDI assay: (1) inoculum preparation; (2–6) microplate preparation; (7) dark control (transfer of 3 *μ*L before illumination); (8) light exposure; (9) transfer of 3 *μ*L after illumination; (10) assessment of the absence or presence of bacterial growth

The MBC was determined by identifying the pipetting point on each dish corresponding to the lowest extract concentration that showed no visible bacterial growth. Figure 3 presents a graphical description of the procedure adopted for the aPDI assay.

## Results and discussion

As shown in Table 1, the extract of *P. rubra* flowers presents phenolic compounds divided into five classes. The flavonol class was predominant, with rutin (quercetin-3-*O*-rutinoside) being the primary compound, with a concentration of 83.96 mg/g, followed by kaempferol-*O*-pentoside-hexoside (4.04 mg/g) and quercetin-3-*O*-glucoside (2.90 mg/g). Among the hydroxycinnamic acids, chlorogenic acid stood out as the main compound, with 13.38 mg/g, while p-coumaric acid glucoside presented 0.59 mg/g. In the iridoid class, *α*-protoplumericin B/C was the most abundant (2.89 mg/g), while caffeoyl-15-demethyl-plumieride was detected in smaller amounts (0.07 mg/g). Regarding phenylpropanoids, only cerberic acid B was identified, at a concentration of 2.95 mg/g. Additionally, in the hydroxybenzoic acid class, 3,4-dihydroxybenzoic acid was present at a concentration of 0.12 mg/g.

**Table 1:** Tentative identification and quantification of phenolic compounds from *P. rubra* flower extract

Peak	Rt (min)	*λ* (nm)	[M–H]$^-$ (m/z)	MS$^2$ (m/z)	Molecular formula	Assignment	Class	Concentration (mg/g)
1	5.37	nd	325.09299	163.04005, 119.05020	C$_{15}$H$_{18}$O$_8$	*p*-Coumaric acid glucoside	Hydroxycinnamic acid	0.59 ± 0.01
2	6.30	nd	153.0194	109.02953	C$_7$H$_6$O$_4$	3,4-Dihydroxybenzoic acid	Hydroxybenzoic acid	0.12 ± 0.01
3	7.63	324	353.08755	191.05614, 179.03532	C$_{16}$H$_{18}$O$_9$	Chlorogenic acid	Hydroxycinnamic acid	13.38 ± 0.46
4	10.95	280	209.04552	191.03503	C$_{11}$H$_{10}$O$_5$	Cerberic acid B	Phenylpropanoid	2.95 ± 0.06
5	13.82	256, 352	741.1882	300.02753, 301.03561	C$_{32}$H$_{38}$O$_{20}$	Quercetin-*o*-rhamnoside-pentoside-hexoside	Flavonol	2.23 ± 0.10
6	15.36	352, 256	595.13037	300.02756, 301.03275	C$_{26}$H$_{28}$O$_{16}$	Quercetin-*o*-pentoside-hexoside	Flavonol	0.67 ± 0.05
7	15.49	356, 256	595.13019	300.02756, 301.03537	C$_{26}$H$_{28}$O$_{16}$	Quercetin-*o*-pentoside-hexoside	Flavonol	2.12 ± 0.04
8	16.82	356, 256	609.14563	300.02750, 301.03525	C$_{27}$H$_{30}$O$_{16}$	Quercetin-*o*-neohesperidoside	Flavonol	2.27 ± 0.15
9	17.08	256, 356	609.14563	301.03247, 300.02750	C$_{27}$H$_{30}$O$_{16}$	Rutin	Flavonol	83.96 ± 1.99
10	17.61	256, 356	739.1733	300.02753, 595.13037, 637.14105, 677.17279	C$_{32}$H$_{36}$O$_{20}$	Quercetin-*o*-hexoside-pentoside-methylglutarate	Flavonol	0.18 ± 0.01
11	18.62	256, 352	463.0000	301.02753	C$_{21}$H$_{20}$O$_{12}$	Quercetin-3-*o*-glucoside	Flavonol	2.90 ± 0.02
12	18.63	256, 353	579.135	284.03247, 285.04034	C$_{26}$H$_{28}$O$_{15}$	Kaempferol-*o*-pentoside-hexoside	Flavonol	4.04 ± 0.03
13	19.02	344, 264	593.15143	284.03565, 285.04047	C$_{27}$H$_{30}$O$_{15}$	Kaempferol-3-*o*-rutinoside	Flavonol	0.08 ± 0.20
14	18.89	296, 320	779.2043	179.03494, 617.15106, 213.05566, 573.16132	C$_{35}$H$_{40}$O$_{20}$	Caffeoyl-15-demethyl-plumieride	Iridoid	0.07 ± 0.01
15	19.59	304	763.2088	213.05573, 169.06587	C$_{35}$H$_{39}$O$_{19}$	*α*-Protoplumericin B or C	Iridoid	2.89 ± 0.02
16	19.93	292	763.20917	213.05566, 169.06586, 163.03999	C$_{35}$H$_{39}$O$_{19}$	*α*-Protoplumericin B or C	Iridoid	0.20 ± 0.01
17	20.53	356, 256	607.13068	300.02753, 301.03531, 463.08804, 505.09875, 545.13043	C$_{27}$H$_{28}$O$_{16}$	Quercetin-*o*-hexoside-dimethylglutarate	Flavonol	0.75 ± 0.04
18	20.92	344, 264	447.0344	285.04047, 284.03262	C$_{21}$H$_{20}$O$_{11}$	Kaempferol-*o*-galactoside	Flavonol	0.09 ± 0.01
19	22.09	344, 264	447.0928	285.03650, 447.09665	C$_{21}$H$_{20}$O$_{11}$	Kaempferol-3-*o*-glucoside	Flavonol	0.59 ± 0.01
20	24.75	348, 264	591.13562	285.04050, 447.09354, 489.10394, 529.13477	C$_{27}$H$_{28}$O$_{15}$	Kaempferol-3-*o*-glucoside-6”-(3-hydroxy-3-methylglutarate)	Flavonol	0.60 ± 0.06
21	30.31	312	601.15631	169.06590, 213.05574, 163.04007	C$_{29}$H$_{30}$O$_{14}$	15-demethyl-plumieride-p-*E*-coumarate	Iridoid	0.13 ± 0.01

The *P. rubra* flowers extract stands out for presenting rutin at a higher concentration. Rutin is considered a potent free radical inhibitor and scavenger, capable of protecting lipids from peroxidation and modulating inflammatory pathways. Its effects include cardiovascular, neuroprotective, antidiabetic, analgesic, gastroprotective, anticancer, and dermatological protection, as well as antiviral, antibacterial, and antispasmodic activities (Casa et al. 2000; Yang et al. 2008; Mittal et al. 2018; Iriti et al. 2023; Bazyar et al. 2023; Forouzanfar et al. 2025).

Studies have shown that the combination of rutin and PS can significantly improve the efficacy of aPDT. This improvement stems from the presence of saccharide groups in rutin, which act to orient PS toward the polysaccharide domains of bacterial cell walls, facilitating PS permeation. Furthermore, in a study using Methylene Blue (MB) as PS, the interaction with rutin alters MB from its dimeric form to the monomeric form, which is more active for aPDT. Thus, rutin acts on both the aPDT process and the chemical stability of PS (Motallebi et al. 2020; Szpakowska et al. 2001).

**Fig. 4:**
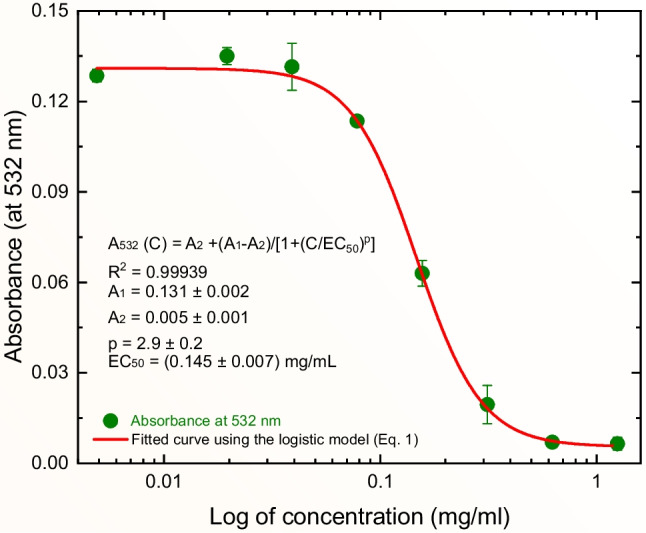
Absorbance at 532 nm as a function of the concentration of *P. rubra* flower extract, subjected to the TBARS assay to evaluate antioxidant activity. The $\textrm{EC}_{50}$ value was obtained by theoretical fitting using a logistic model (Eq. 1). The parameter p reflects the sensitivity of the extract’s antioxidant activity to variations in concentration

Figure 4 shows the results from the antioxidant activity assay (TBARS) by the absorbance values at 532 nm as a function of the concentration of the extract from the flowers of *P. rubra* together with the theoretical adjustment (red line) using a logistic model given by Eq. 1, which allowed obtaining the $\textrm{EC}_{50} = (0.145 \pm 0.007) \, \mathrm {mg/mL}$ and a sensitivity parameter $\textrm{p} = 2.9 \pm 0.2$

For p = 1, a typical sigmoid response is observed, in which the antioxidant effect increases progressively and continuously with extract concentration. When the p parameter assumes values less than 1 (p < 1), the relationship between absorbance and extract concentration presents a more gradual transition, indicating that increasing concentration promotes a less uniform response. This behavior may suggest the presence of multiple antioxidant compounds with different reducing potentials or low homogeneity in the antioxidant process (Sorribas et al. 2007; Noel et al. 2018).

On the other hand, p > 1 indicates a more abrupt transition, in which slight variations in concentration around the $\textrm{EC}_{50}$ result in significant changes in antioxidant activity. This behavior suggests the occurrence of cooperative or synergistic mechanisms between the antioxidant compounds present in the extract, which may be associated with the *P. rubra* extract, given the observed value for the p parameter of $2.9\,\pm \, 0.2$ (Sorribas et al. 2007; Noel et al. 2018).

**Fig. 5:**
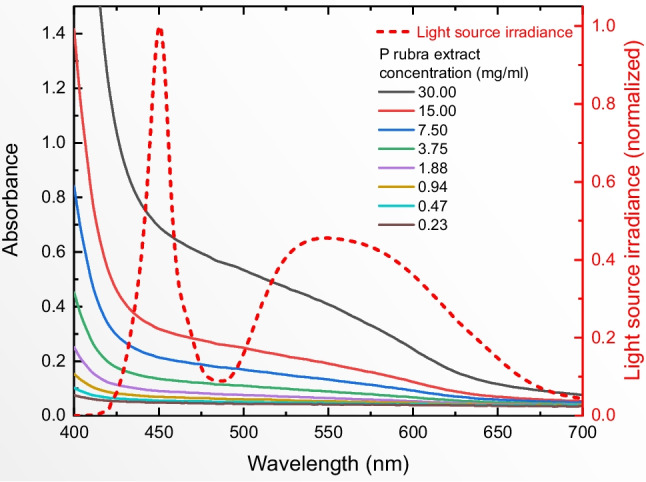
UV-VIS spectra of *P. rubra* flower extract in aqueous solution under different concentrations (solid lines) and irradiance of the light source used in the aPDI assay (red dashed line)

Figure 5 shows the absorbance spectra of *P. rubra* flower extracts at different concentrations, ranging from 30.00 to 0.23 mg/mL. The extract exhibits a broad optical absorption range, extending from approximately 400 to 630 nm. Based on this spectral behavior, we chose to use a light source composed of a set of LEDs emitting white light, characterized by two broad bands centered around 450 and 550 nm, in the aPDI assays.

The choice of white light, in addition to presenting an adequate spectral match with the extract’s absorption region, offers practical and economic advantages, since such sources are widely commercially available, low-cost, and do not require the use of lasers and optical filters, characteristics that make them particularly attractive for industrial-scale applications.

The antimicrobial activity assay, in the dark, was performed to verify whether the extract exhibits antimicrobial activity (in the dark) and at what MIC and MBC values this occurs. The results show that, within the investigated concentration range of 0.1 to 100.00 mg/mL, the extract did not exhibit antimicrobial activity. That is, both MIC and MBC were considered greater than 100 mg/mL indicating that the *P. rubra* extract does not exhibit antimicrobial activity in the dark, which is a desirable characteristic in a PS. Based on this result, aPDI assays were performed to investigate different concentrations of the extract obtained by serial dilution, starting from 50.00 mg/mL.

The methodology used in the aPDI assay did not aim to quantify microbial reduction, but rather to determine the minimum extract concentration capable of exhibiting bactericidal action, as well as the corresponding optical fluence values. To this end, the images of the Petri dishes were analyzed to identify the pipetting points that showed no visible signs of microbial growth.

**Fig. 6:**
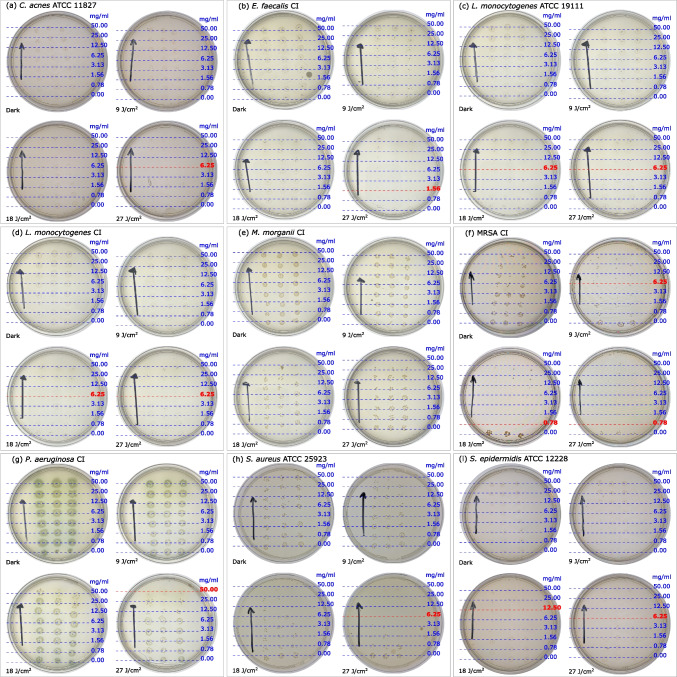
Photographs of Petri dishes for different bacterial strains and optical fluence values (Dark = 0 J/cm$^2$). The arrow drawn on the background of each plate indicates the direction of increasing *P. rubra *extract concentration, ranging from 0 to 50.00 mg/mL

Figure 6 shows the images of the Petri dishes for the different bacterial strains, at the four light doses used: 0 $\mathrm {J/cm}^{2}$ (dark control), 9 $\mathrm {J/cm}^{2}$, 18 $\mathrm {J/cm}^{2}$, and 27 $\mathrm {J/cm}^{2}$, along with the respective extract concentrations corresponding to each pipetting point.

During the analysis of the Petri dish images, care was taken to distinguish between the marks left by the micropipette tip’s contact with the solid medium and possible signs of bacterial growth. The distinction between these two types of marks is easily discernible: while the tip’s contact produces a well-defined and localized spot, bacterial growth manifests itself as a diffuse halo, with a larger area and irregular distribution. Therefore, only pipetting points that did not show any visible evidence of bacterial colonies were considered free of multiplication and used for the determination of MBC.

In the aPDI assay, including the dark control, it was observed that the P*. rubra* flower extract showed no antimicrobial activity at concentrations up to 50.00 mg/mL. This result was expected, since similar behavior had been observed in the antimicrobial activity assay performed in the absence of light.

When applied at an optical fluence of 9 $\mathrm {J/cm^2}$, the extract was found to inactivate only the MRSA CI strain, presenting a MBC of 6.25 mg/mL. At an optical fluence of 18 $\mathrm {J/cm^2}$, the MBC for MRSA CI decreased to 0.78 mg/mL, while the MBC for *L. monocytogenes* (ATCC and CI) were 6.25 mg/mL, and for *S. epidermidis*, 12.50 mg/mL.

Under an optical fluence of 27 $\mathrm {J/cm^2}$, inactivation of all bacterial strains tested was observed, except for *M. morganii*. Under these conditions, the MBC was 6.25 mg/mL for* C. acnes*, *L. monocytogenes* (ATCC and CI), *S. aureu*s, and *S. epidermidis*; 1.56 mg/mL for *E. faecalis*; 0.78 mg/mL for MRSA CI; and 50.00 mg/mL for *P. aeruginosa*.

**Fig. 7:**
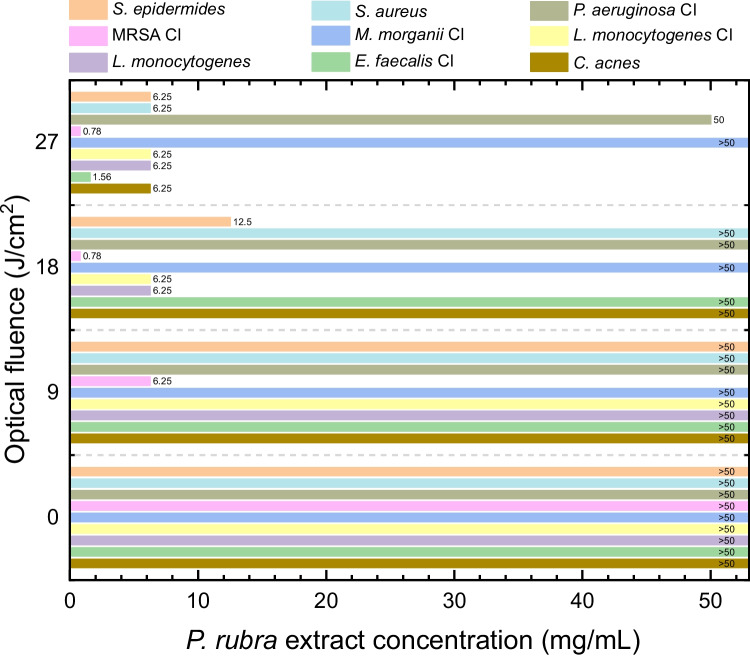
MBC of *P. rubra* flower extract as a function of the optical fluence applied to different bacterial strains. Values greater than 50 mg/mL indicate that the extract did not present aPDI within the range of concentrations and optical fluences tested

Figure 7 presents a comparative graph of the MBC for each bacterial strain under different optical fluence regimes, as determined from the analysis of Petri dish images.

The results obtained in the aPDI assay demonstrated that *P. rubra* extract exhibits photodynamic inactivation effects against various bacteria. The exceptions were *M. morganii*, which was not inactivated under any of the tested conditions, and *P. aeruginosa*, which was inactivated only at the highest optical fluence and extract concentration. This is because, unlike the others, the two bacteria are Gram-negative characterized by a dual-membrane cell wall structure which makes them more resistant to aPDI than Gram-positive bacteria (Rapacka-Zdonczyk et al. 2021).

## Conclusion

The extract from the flowers of *P. rubra* is a rich source of phenolic compounds, with rutin being the main constituent (83.96 mg/g). This phytochemical composition confers the extract a high antioxidant potential, evidenced by the cooperative behavior between its components ($\mathrm {p = 2.9}$).

The absence of antimicrobial activity in the dark (MIC/MBC > 100 mg/mL) is a crucial characteristic for a PS. When exposed to white light (an affordable and accessible source of irradiation), the extract exhibited significant aPDI against Gram-positive bacteria, including multidrug-resistant strains (MRSA CI, MBC = 0.78 mg/mL at 18 $\mathrm {J/cm^2}$). The efficacy was fluence-dependent, inactivating most strains at 27 $\mathrm {J/cm^2}$, except the most resistant Gram-negative strains.

In summary, the extract from the flowers of *P. rubra* is a promising natural PS, combining a superior phytochemical profile, inactivity in the dark, and potent photodynamic bactericidal action, validating its potential for applications in aPDI.

## Limitations and future perspectives

Future studies should include in vivo models to validate the efficacy observed under in vitro conditions and broaden the biological relevance of the results. The incorporation of three-dimensional models and biofilm-based systems is recommended, as they more accurately reflect microbial organization and tolerance mechanisms associated with persistent infections. Additionally, future studies should standardize the process for obtaining *P. rubra* extract and systematically evaluating the influence of environmental factors related to the region and cultivation conditions, such as climate, edaphoclimatic characteristics, and plant development stage, on its photodynamic activity. Finally, expanding the number and diversity of microbial strains evaluated will allow for a more comprehensive understanding of photodynamic efficacy in the face of genetic and phenotypic heterogeneity and distinct resistance mechanisms of clinical relevance.

## Data Availability

No datasets were generated or analysed during the current study.

## References

[CR1] Acevedo-Rodríguez P, Strong MT (2012) Catalogue of seed plants of the West Indies. Smithsonian Institution, Washington, DC

[CR2] Aebisher D, Przygórzewska A, Bartusik-Aebisher D (2024) Natural photosensitizers in clinical trials. Appl Sci 14:8436

[CR3] Athirojthanakij W, Rashidinejad A (2024) Optimizing catechin extraction from green tea waste: Comparative analysis of hot water, ultrasound-assisted, and ethanol methods for enhanced antioxidant recovery. Food Science & Nutrition 12:5121–5130 39055189 10.1002/fsn3.4161 PMC11266887

[CR4] Aziz B et al (2023) An overview of potential natural photosensitizers in cancer photodynamic therapy. Biomedicines 11:224 36672732 10.3390/biomedicines11010224 PMC9855789

[CR5] Bazyar H, Moradi L, Zaman F, Zare Javid A (2023) The effects of rutin flavonoid supplement on glycemic status, lipid profile, atherogenic index of plasma, brain-derived neurotrophic factor (bdnf), some serum inflammatory, and oxidative stress factors in patients with type 2 diabetes mellitus: a double-blind, placebo-controlled trial. Phytotherap Res 37:271–284 10.1002/ptr.7611 36101997

[CR6] Bihani T (2021) Plumeria rubra l.– a review on its ethnopharmacological, morphological, phytochemical, pharmacological and toxicological studies. J Ethnopharmacol 264:113291 32841700 10.1016/j.jep.2020.113291

[CR7] Byamukama R, Namukobe J, Jordheim M, Øyvind MA, Kiremire BT (2011) Anthocyanins from ornamental flowers of red frangipani, plumeria rubra. Sci Hortic 129:840–843

[CR8] Calva J, Celi J, Benítez A (2024) Analysis of the volatile and enantiomeric compounds emitted by plumeria rubra l. flowers using hs-spme–gc. Plants 13 10.3390/plants13172367 PMC11397236 39273851

[CR9] Casa CL, Villegas I, Alarcón de la Lastra C, Motilva V, Martín Calero MJ (2000) Evidence for protective and antioxidant properties of rutin, a natural flavone, against ethanol induced gastric lesions. J Ethnopharmacol 71:45–53 10.1016/s0378-8741(99)00174-9 10904145

[CR10] Castano AP, Demidova TN, Hamblin MR (2004) Mechanisms in photodynamic therapy: Part one - photosensitizers, photochemistry and cellular localization. Photodiagn Photodyn Ther 1:279–293 10.1016/S1572-1000(05)00007-4 PMC4108220 25048432

[CR11] Christodoulou MC et al (2022) Spectrophotometric methods for measurement of antioxidant activity in food and pharmaceuticals. Antioxidants 11 10.3390/antiox11112213 PMC9686769 36358583

[CR12] Cui L, Ma Z, Wang D, Niu Y (2022) Ultrasound-assisted extraction, optimization, isolation, and antioxidant activity analysis of flavonoids from astragalus membranaceus stems and leaves. Ultrason Sonochem 90:106190 36215890 10.1016/j.ultsonch.2022.106190 PMC9554832

[CR13] Dougherty TJ et al (1998) Photodynamic therapy. J Natl Cancer Inst 90:889–905 9637138 10.1093/jnci/90.12.889 PMC4592754

[CR14] Ferreira CP, da Costa de Lima M, da Silva JG, Peixoto Araujo NM (2024) Nutritional composition, phenolic compounds and biological activities of selected unconventional food plants. Food Res Intern 191:114643 10.1016/j.foodres.2024.114643 39059900

[CR15] Fiegler-Rudol J, Łopaciński M, Los A, Skaba D, Wiench R (2025) Riboflavin-mediated photodynamic therapy in periodontology: A systematic review of applications and outcomes. Pharmaceutics 17 10.3390/pharmaceutics17020217 PMC11859475 40006584

[CR16] Forouzanfar F, Sahranavard T, Tsatsakis A, Iranshahi M, Rezaee R (2025) Rutin: a pain-relieving flavonoid. Inflammopharmacology 33:1289–1301 39961908 10.1007/s10787-025-01671-8

[CR17] Ghani MA, Barril C, Bedgood DR, Prenzler PD (2017) Measurement of antioxidant activity with the thiobarbituric acid reactive substances assay. Food Chem 230:195–207 28407901 10.1016/j.foodchem.2017.02.127

[CR18] Gilman EF, Watson DG (1994) Plumeria rubra frangipani. Fact Sheet ST-490. Enviromental Holticulture Departement, Florida Cooperative Extension Service, Institute of Food and Agriculture Sciences, University of Florida

[CR19] Gonçalves GCP et al (2024) A green method for anthocyanin extraction from clitoria ternatea flowers cultivated in southern brazil: Characterization, in vivo toxicity, and biological activity. Food Chem 435:137575 37776651 10.1016/j.foodchem.2023.137575

[CR20] Hamblin MR (2016) Antimicrobial photodynamic inactivation: a bright new technique to kill resistant microbes. Curr Opin Microbiol 33:67–73 27421070 10.1016/j.mib.2016.06.008 PMC5069151

[CR21] Hamburger MO, Cordell GA, Ruangrungsi N (1991) Traditional medicinal plants of thailand xvii biologically active constituents of plumeria rubra. J Ethnopharmacol 33:289–292 1921428 10.1016/0378-8741(91)90091-q

[CR22] Henderson BW, Dougherty TJ (1992) How does photodynamic therapy work? Photochem Photobiol 55:145–157 1603846 10.1111/j.1751-1097.1992.tb04222.x

[CR23] Herculano LS et al (2026) Azure a as a promising candidate for antimicrobial photodynamic therapy of acne-related bacteria. Photochem Photobiol Sci 10.1007/s43630-026-00861-9 41678115

[CR24] Iriti M, Varoni EM, Vitalini S (2023) Bioactive compounds in health and disease – focus on rutin. Funct Foods Health Dis 6:235–242

[CR25] Isa SSPM, Ablat A, Mohamad J (2018) The antioxidant and xanthine oxidase inhibitory activity of plumeria rubra flowers. Molecules: J Synth Chem Natural Prod Chem 23 10.3390/molecules23020400 PMC6017381 29438299

[CR26] Kalantri M, Aher AN (2016) Review on traditional medicinal plant: *plumeria rubra*. J Medicin Plants Stud 4:204–207

[CR27] Kumar K, Srivastava S, Sharanagat VS (2021) Ultrasound assisted extraction (uae) of bioactive compounds from fruit and vegetable processing by-products: A review. Ultrason Sonochem 70:105325 32920300 10.1016/j.ultsonch.2020.105325 PMC7786612

[CR28] Leal M, Alves R, Hanazaki N (2018) Knowledge, use, and disuse of unconventional food plants. J Ethnobiol Ethnomed 14:6 29343263 10.1186/s13002-018-0209-8 PMC5773074

[CR29] Malaspina P et al (2024) Beyond the scent: New evidence about micromorphological, phytochemical and biological features of Plumeria rubra ‘tonda palermitana’ (apocynaceae). Plants 13:2479 39273963 10.3390/plants13172479 PMC11397171

[CR30] Milião GL et al (2022) Unconventional food plants: Nutritional aspects and perspectives for industrial applications. Future Foods 5:100124

[CR31] Mittal R, Kumar A, Singh D, Bishnoi M, Nag T (2018) Ameliorative potential of rutin in combination with nimesulide in stz model of diabetic neuropathy: targeting nrf2/ho-1/nf-kb and cox signalling pathway. Inflammopharmacology 26:755–768 29094308 10.1007/s10787-017-0413-5

[CR32] Motallebi M, Khorsandi K, Sepahy AA, Chamani E, Hosseinzadeh R (2020) Effect of rutin as flavonoid compound on photodynamic inactivation against p. aeruginosa and s. aureus. Photodiagnos Photodynam Therap 32:102074 10.1016/j.pdpdt.2020.102074 33137496

[CR33] Nayak P, Sahani S, Samal H et al (2025) Photodynamic therapy: Advancement in therapeutic and cosmetic application for targeted treatment: A review. Biomed Mater Dev 3:1150–1166

[CR34] Noel ZA, Wang J, Chilvers MI (2018) Significant influence of ec50 estimation by model choice and ec50 type. Plant Dis 102:708–714 30673399 10.1094/PDIS-06-17-0873-SR

[CR35] Pang D et al (2025) Antimicrobial mechanism of typical microbial preservatives and its potentiation strategy by photodynamic inactivation: A review. Food Microbiol 132:104833 40683717 10.1016/j.fm.2025.104833

[CR36] Qureshi S, Rehan Z, Ao A, Mukovozov I (2024) Photodynamic therapy in acne vulgaris: A systematic review. J Cutan Med Surg 29:69–73 39552358 10.1177/12034754241291031 PMC11829500

[CR37] Rapacka-Zdonczyk A et al (2021) Factors determining the susceptibility of bacteria to antibacterial photodynamic inactivation. Front Med 8:642609 10.3389/fmed.2021.642609 PMC8149737 34055830

[CR38] Rojas-Sandoval J (2024) Plumeria rubra (red frangipani). CABI Compendium. Accessed 8 Oct 2025

[CR39] Sethi S, Rathod VK (2025) Recent advances in ultrasound-assisted extraction of natural products using novel solvents: a mini-review. Curr Opin Chem Eng 48:101132

[CR40] Sharma G et al (2011) Phytochemical constituents, traditional uses, and pharmacological properties of the genus plumeria. Chem Biodiver 8:1357–1369

[CR41] Silva JVM, Santos AS, Pereira GA, Chisté RC (2023) Ultrasound-assisted extraction using ethanol efficiently extracted carotenoids from peels of peach palm fruits (bactris gasipaes kunth) without altering qualitative carotenoid profile. Heliyon 9:e14933 37089291 10.1016/j.heliyon.2023.e14933 PMC10114151

[CR42] Sorribas A, Hernández-Bermejo B, Vilaprinyo E, Alves R (2007) Cooperativity and saturation in biochemical networks: A saturable formalism using taylor series approximations. Biotechnol Bioeng 97:1259–1277 17187441 10.1002/bit.21316

[CR43] Szpakowska M, Lasocki K, Grzybowski J, Graczyk A (2001) Photodynamic activity of the haematoporphyrin derivative with rutin and arginine substituents (hpd-rut2- arg2) against staphylococcus aureus and pseudomonas aeruginosa. Pharmacol Res 44:243–246 11529692 10.1006/phrs.2001.0855

[CR44] Vahl A (2012) Flora of panama (wfo). Tropicos. org

[CR45] Wang X et al (2023) Polyphenolic natural products as photosensitizers for antimicrobial photodynamic therapy: recent advances and future prospects. Front Immunol 14:1275859 38022517 10.3389/fimmu.2023.1275859 PMC10644286

[CR46] Yang J, Guo J, Yuan J (2008) In vitro antioxidant properties of rutin. LWT Food Sci Technol 41:1060–1066

[CR47] Zhou X et al (2024) Research progress of natural product photosensitizers in photodynamic therapy. Planta Med 90:368–379 38423033 10.1055/a-2257-9194

